# Acute Kidney Injury in Pediatric Treated with Vancomycin and Piperacillin-Tazobactam in Tertiary Care Hospital

**DOI:** 10.1155/2018/9256528

**Published:** 2018-12-16

**Authors:** Mohammed Al Nuhait, Laila C. Abu Esba, Khalid Al Harbi, Meshary Al Meshary, Rami T. Bustami

**Affiliations:** ^1^King Abdulaziz Medical City, Pharmaceutical Care Services, Ministry of National Guard Health Affairs, Riyadh, Saudi Arabia; ^2^College of Pharmacy, King Saud Bin Abdul-Aziz University for Health Sciences (KSAU-HS), Ministry of National Guard Health Affairs, Riyadh, Saudi Arabia; ^3^Prince Sultan Military Medical City, Department of Pharmacy, Riyadh, Saudi Arabia

## Abstract

**Background:**

Vancomycin and piperacillin-tazobactam (PTZ) are commonly used as empirical therapy for patients with health care associated infections. Vancomycin has been recognized as a nephrotoxic agent and in a few cases in the literature PTZ has been associated with interstitial nephritis nevertheless; the combination of these agents has routinely been used for many years. However, there have been some observational studies that showed high rates of acute kidney injury (AKI) in patients receiving vancomycin and PTZ concomitant treatment compared to patients receiving vancomycin alone. The incidence of AKI in adult patients receiving vancomycin and PTZ concomitant treatment was reported in these studies to be relatively high. Similar studies in pediatric patients are lacking.

**Method:**

We conducted a single center retrospective chart review of 248 pediatric patients receiving one of the following treatments: vancomycin alone 36 patients, vancomycin/PTZ 62 patients, vancomycin/ceftazidime 99 patients, and vancomycin/ceftriaxone 51 patients.

**Result:**

Our results showed a low incidence of AKI in patients on vancomycin/PTZ concomitant treatment where overall incidence was only (4.8%) three cases and only one of them (2.0%) in a patient receiving the vancomycin/ceftriaxone concomitant treatment. No cases of AKI present in patients receiving vancomycin with ceftazidime or vancomycin alone. There were no statistically significant differences between the four treatment groups in terms of AKI incidence, vancomycin trough, and use of nephrotoxins.

**Conclusion:**

Overall, the incidence of AKI was low in our study sample with no statistically significant increased risk when PTZ was used in combination with vancomycin in a pediatric population. However, further investigation with an equal larger sample size is needed to confirm our findings.

## 1. Background

Vancomycin is a glycopeptide antibacterial drug that is commonly used in the hospital setting for treatment of gram-positive infections and infections caused by methicillin-resistant Staphylococcus aureus (MRSA) [1]. Piperacillin-tazobactam (PTZ) is a combination of piperacillin and the b-lactamase inhibitor tazobactam. Piperacillin-tazobactam has a broad-spectrum antibacterial activity that covers aerobic gram-positive, gram-negative, and anaerobic bacteria but does not cover MRSA [2, 3].

Vancomycin and PTZ are commonly used as empirical therapy for patients with healthcare-associated infections as they are active against both methicillin-resistant Staphylococcus aureus (MRSA) and Pseudomonas aeruginosa [4]. Vancomycin has been recognized as a nephrotoxic agent, and in a few cases in the literature PTZ has been associated with interstitial nephritis [5–7].

In recent years, there have been some retrospective studies that showed high rates of acute kidney injury (AKI) in patients receiving vancomycin and piperacillin-tazobactam concomitant therapy which are higher than those reported for patients receiving vancomycin alone [8–12]. A retrospective cohort study at Duke University hospital reported a significantly lower incidence of AKI in patients receiving vancomycin (8.1%) compared to those treated with vancomycin and PTZ (16.3%) [8]. Another retrospective cohort study conducted at the University of Florida's Health Science Center found that the incidence of AKI was significantly higher in patients receiving PTZ with vancomycin (34.8%) compared to patients receiving cefepime with vancomycin (12.5%) [9].

Several other studies on different combination of medications showed that AKI rates were significantly higher in patients receiving vancomycin plus PTZ than vancomycin plus cefepime or other antibiotic medications [10–12]. In contrast, a number of studies reported similar AKI risk in these groups who received combination treatment [13, 14]. In summary, the incidence of AKI in adult patients receiving a combination of vancomycin and PTZ was relatively high, ranging from 15 to 38% [8–12, 15, 16], as the incidence of AKI and risk factors are not yet established well for pediatric patients, where only a few studies and case reports are available in the literature [15, 16]. This research aimed to shed light on the incidence and risk factors of AKI in pediatric patients treated with vancomycin and PTZ compared to vancomycin alone or in combination with a cephalosporin that is commonly used in our hospital.

## 2. Methods

This is a single-center retrospective cohort study to evaluate the incidence and risk of AKI in pediatric patients treated with vancomycin with PTZ, ceftazidime, or ceftriaxone versus vancomycin monotherapy, at King Abdullah Children Specialized Hospital (KASCH) which is the first specialist children's hospital in the Kingdom of Saudi Arabia, occupying 192,000 m^2^, over 10 levels with a total bed capacity of 600 beds. The study period was from September 2015 to December 2016. Study participants were identified using our electronic health care system, to identify patients who were treated with vancomycin during the study period. AKI was defined using the Kidney Disease: Improving Global Outcomes (KDIGO) criteria; as an increase in serum creatinine (SCr) by ≥ 0.3 mg/dl (≥26.5 *μ*mol/l) within 48 hours; or as an increase in SCr level by 50% or higher from baseline. Inclusion criteria were patients aged one month to 14 years and had received vancomycin with PTZ, ceftazidime or ceftriaxone concomitantly or vancomycin monotherapy for ≥ 48 hours. Exclusion criteria were (A) patients currently on dialysis, having a history of chronic kidney disease (CKD) (stage III or higher) or structural kidney disease (e.g., one kidney, kidney transplant, kidney tumor); (B) diagnosis of kidney injury due to causes other than the therapy; (C) ICU patients; (D) receiving therapy less than 48 hours; (E) incomplete lab data (SCr); (F) receiving local routes of vancomycin like intraperitoneal, eye drops, or oral vancomycin;(G) neonates less than 1 month old.

Pediatric patients admitted to KACSH wards and who met the above-specified inclusion and exclusion criteria were included.

The following patient demographic and clinical data were collected: age, height, weight, gender, dose of vancomycin, PTZ, ceftazidime and ceftriaxone, laboratory data (SCr and blood urea nitrogen), treatment indication, and initial vancomycin serum trough concentrations.

Statistical analysis: descriptive statistical analyses were performed for the study participants. Continuous variables were summarized using mean ± SD, median, and interquartile range (IQR). Proportions were used for categorical variables. The four medication groups were compared in terms of baseline demographic and clinical characteristics as well as incidence of AKI. Comparisons were made using the one-way ANOVA or Kruskal-Wallis test for continuous variables and the chi-square test for categorical variables. A logistic regression model was utilized to estimate and adjust odds ratio of AKI in the four groups. The model was adjusted for several baseline demographic and clinical characteristics. Statistical significance was considered at p<0.05. All statistical analyses were performed using SPSS 21.0 [Release 21.0.0.0, IBM, USA].

## 3. Results

A total of 737 patients who received vancomycin during the study period were screened for inclusion in the study. A total of 248 met the specified inclusion criteria for our study. Descriptive statistics of the demographic and baseline characteristics of the patients are presented in Table 1. The number of patients in each treatment group was as follows: vancomycin alone 36 (14.5%), vancomycin and PTZ 62 (25.0%), vancomycin and ceftazidime 99 (39.9%), and vancomycin and ceftriaxone 51 (20.6%). Average ± SD age was 5.3 ±3.4 years with 54% males. Indications for treatment were documented to be empirical in 32%, pneumonia 15%, fever 13%, URTI 9%, sepsis 7%, febrile neutropenia 4%, meningitis 4%, and 26% had other diagnoses. Underlying conditions of the patients were 12% congenital heart disease, 18.5% asthma, and 14.5% seizures. Around 30% of patients used other nephrotoxic medications. The average length of stay (LOC) before therapy was 6.7 days (SD= 19.9).

The distribution of cultures is presented in Table 2. Only 84 (34%) patients had a positive culture; the majority of them had* Staphylococcus aureus*,* Pseudomonas aeruginosa*,* Klebsiella pneumonia*,* Candida albicans, *or* Escherichia coli*.

Table 3 shows descriptive statistics for antibiotics (vancomycin, PTZ, ceftazidime, and ceftriaxone) dose and frequency as well as total days of therapy. The table also includes descriptive follow-up data. Descriptive statistics for outcomes (AKI and total LOS) are also provided in Table 4.

Results from comparing the four treatment groups are shown in Table 4.

There were no statistically significant differences between the four treatment groups in terms of AKI incidence, vancomycin trough, use of other nephrotoxins, or duration of therapy (p>0.05). As Table 4 illustrates, the treatment groups differed significantly in terms of vancomycin dose and total LOS. Patients receiving vancomycin plus PTZ or vancomycin plus ceftriaxone had a statistically significant higher vancomycin dose than those in the other two groups (p = 0.019). Although they seemed to have received higher doses of vancomycin than the other two groups, we did not see an increased risk of AKI in those groups of patients. Also, patients in the vancomycin plus PTZ group had the longest LOS. So, despite higher doses and longest LOS, no significant increased risk was seen.

## 4. Discussion

Vancomycin is used usually concomitantly with other antibacterial drugs like beta-lactams; in recent years there have been some retrospectives studies indicating that using vancomycin with PTZ is associated with an increased risk of nephrotoxicity compared to vancomycin alone or vancomycin with other beta-lactams [8–12]. Most of these studies were conducted in adults while only a few studies and case reports were conducted in pediatric patients.

In general, there are many factors that might increase the risk of nephrotoxicity in patients receiving vancomycin plus PTZ beside the concomitant use of this combination, such as higher doses of vancomycin or PTZ, a high vancomycin trough level, and/or concomitant use of other nephrotoxic medications [8, 17–19].

PTZ in combination with vancomycin tends to be a more likely treatment selection in sicker patients as seen in our study where a significantly higher LOC was seen in this combination group. In our study, the overall incidence of AKI was low (1.4%), 4 cases, where 3 cases (4.8%) occurred in the vancomycin plus PTZ group and one case in the vancomycin plus ceftriaxone group, and no cases of AKI were seen in the vancomycin monotherapy or vancomycin plus ceftazidime groups. Nevertheless, the difference between the four groups in AKI incidence, vancomycin trough, use of other nephrotoxic drugs, or duration of therapy did not reach a statistical significance. It is worth noting that there was no increased risk of AKI in the vancomycin plus PTZ combination group despite statistically significantly higher doses of vancomycin used in this group.

Table 5 shows further assessment of the AKI cases revealed that two of the three patients with nephrotoxicity in the vancomycin plus PTZ group were on other nephrotoxic medications (i.e., gentamicin, acyclovir). And that the only case that occurred in a patient who received vancomycin and ceftriaxone had an elevated serum vancomycin trough (27.5 mg/L). Therefore, our findings confirm the notion that the increased risk of AKI is multifactorial rather than a risk solely associated with the use of this combination and that the concern of AKI possibly not on the top of the list when weighing the benefit versus the risk in the decision of selecting this combination.

In comparison, in our study the incidence of AKI was lower than that reported in other studies. In a study conducted at a single center by McQueen, AKI developed in 3 of 79 patients in the vancomycin group alone and in 25 of 106 patients on vancomycin plus PTZ and they include patients with underlying renal dysfunction in their study unlike ours [20], while on other larger retrospective multicenter study released this year by Downes, the incidence of AKI among those who received vancomycin plus PTZ combination therapy was 117 of 1009 patients (11.7%) and 40 of 906 (4.4 %) in patients who received vancomycin plus other beta-lactam (ceftazidime, cefepime, and meropenem/imipenem) [21].

Our KASCH clinical pharmacy team preforms daily rounds and provides therapeutic drug monitoring (TDM) services for all our pediatric patients, good TDM has been linked to better patient outcome and a reduction in incidents of nephrotoxicity related to vancomycin, and our results applaud on their efforts and reflect their vigilance in monitoring our patients [22–24].

Finally, our study has several limitations that must be addressed. The study was retrospective and had a small sample size. An equal larger sample size is needed to confirm our findings.

## 5. Conclusion

Based on our study, the incidence of AKI was low in our study sample with no statistically significant increased risk when PTZ was used in combination with vancomycin in pediatric population. However, further investigation with an equal larger sample size is needed to confirm our findings.

## Figures and Tables

**Table 1:** Profile of patients, baseline characteristics. N = 248.

**Factor**	**Number (**%**)**	**Factor**	**Mean ± SD** **Median (IQR)**
**Gender**		**Age (years)**	5.3 ± 3.4
Male	134 (54.0%)		5 (2-7)
Female	114 (46.0%)		
**Treatment**		**Weight (kg)**	18.0 ± 12.3
Vancomycin alone	36 (14.5%)		16.0 (9.8-22.0)
Vancomycin and PTZ	62 (25.0%)		
Vancomycin plus Ceftazidime	99 (39.9%)		
Vancomycin plus Ceftriaxone	51 (20.6%)	**Height (cm)**	100.1 ± 28.0
			104 (79-119)
**Indication of Antibiotics **			
Empirical	79 (31.9%)	**Creatinine (mg/dL**)	0.45 ± 0.1
Pneumonia	37 (14.9%)		0.44 (0.38-0.5)
Fever	33 (13.3%)		
URTI	23 (9.3%)		
Sepsis	17 (6.9%)	**BUN (mg/dL**)	10.1 ± 5.3
Other*∗*	59 (23.7%)		8.7 (6.2-13.7)
Underlying conditions			
**History of G6PD deficiency**	6 (2.4%)	**WBC (x 10** ^**9**^ **/L**)	10.9 ± 7.3
**Trauma**	5 (2.0%)		10.0 (5.4-15.0)
**GERD**	14 (5.6%)		
**Cerebral Palsy**	9 (3.6%)	**LOS Before Therapy** (days)	6.7 ± 19.9
**Sickle Cell Disease**	15 (6.0%)		0 (0-4)
**Congenital Heart Disease**	31 (12.5%)		
**Asthma**	46 (18.5%)		
**Seizures**	36 (14.5%)	**Use of concomitant nephrotoxins** **∗** **∗**	76 (30.6%)
**Diabetes**	2 (0.8%)		
**History of Malignancy**	20 (8.1%)		

*∗* Other: febrile neutropenia, meningitis, UTI, bacteremia, skin infection, and trauma.

*∗∗* Aminoglycoside, furosemide, amphotericin B, colistin, ACEIs, ARBs, NSAID, tacrolimus, acyclovir, and cyclosporine.

URTI: upper respiratory tract infection, G5PD: Glucose-6-Phosphate Dehydrogenase, GERD: gastroesophageal reflux disease, BUN: blood urea nitrogen, and LOS: length of stay.

**Table 2:** Culture distribution. N = 248.

**Type**	**N**	**Percent**
*Staphylococcus aureus*	22	8.9%
*Pseudomonas aeruginosa*	13	5.2%
*Klebsiella pneumoniae*	9	3.6%
*Candida albicans*	6	2.4%
*Escherichia coli*	5	2.0%
*MRSA*	4	1.6 %
Other	25	10.1%
*None*	*164*	*66.2*%

Other: Acinetobacter baumannii, S. pneumoniae, Viridans streptococci, Staphylococcus epidermidis, Haemophilus influenzae, Candida parapsilosis, Enterococcus faecalis, Enterococcus faecium, Enterobacter asburiae, Enterobacter cloacae, HSV, Influenza virus B, RSV, Serratia plymuthica, Shigella flexneri.

**Table 3:** Doses, frequency, and follow-up measures.

**Factor**		
**Vancomycin dose (mg/kg/day) **	Mean ± SD	45.8 ± 14.1
	Median (IQR)	45.0 (38.0-58.0)
**Vancomycin frequency **n (%)		
Every 6 hours		172 (69.4%)
Every 8 hours		70 (28.2%)
Every 12 hours		4 (1.6%)
Every 24 hours		2 (0.8%)
**Initial vancomycin trough level (mg/L) **	Mean ± SD	9 ± 5.2
	Median (IQR)	8.1 (4.9-11.7)
**Piperacillin-tazobactam dose (mg/kg/dose) **	Mean ± SD	95.8 ± 24.5
	Median (IQR)	100.0 (89.5-100.0)
**Piperacillin-tazobactam frequency **n (%)		
Every 6 hours		5 (8.1%)
Every 8 hours		57 (91.9%)
**Ceftazidime dose (mg/kg/dose) **	Mean ± SD	45.8 ± 9.2
	Median (IQR)	50.0 (49.0-50.0)
**Ceftazidime frequency **n (%)		
Every 8 hours		99 (100%)
**Ceftriaxone dose (mg/kg/dose) **	Mean ± SD	61.2 ± 31.0
	Median (IQR)	50.0 (45.0-71.3)
**Ceftriaxone frequency **n (%)		
Every 12 hours		10 (19.6%)
Every 24 hours		41 (80.4%)
**WBC (x 10** ^**9**^ **/L**) **48 hours **	Mean ± SD	8.5 ± 5.2
	Median (IQR)	8.0 (5.0-11.4)
**Days of therapy **	Mean ± SD	7.2 ± 7.2
	Median (IQR)	5.0 (4.0-8.0)

**Table 4:** Outcomes (AKI and LOS) and use of concomitant nephrotoxins, vancomycin trough, daily vancomycin dose, and duration of therapy in overall and by treatment. N = 248.

**Groups**					
**Factor**	**All** **Patients** (n=248)	**Vancomycin** (n=36)	**Vancomycin** **+** **PTZ** (n=62)	**Vancomycin** **+** **Ceftazidime** (n=99)	**Vancomycin** **+** **Ceftriaxone** (n=51)
**AKI cases**	**4 (1.6**%**)**	**0 (0.0**%**)**	**3 (4.8**%**)**	**0 (0.0**%**)**	**1 (2.0**%**)**
**Use of concomitant nephrotoxic medication**	**76 (30.6**%**)**	**10 (27.8**%**)**	**21 (33.9**%**)**	**33 (33.3**%**)**	**12 (23.5**%**)**
**Duration of therapy (days) **					
Mean ± SD	**7.2 ± 7.2**	**9.1 ± 13.9**	**7.1 ± 4.9**	**7.4 ± 5.9**	**5.8 ± 4.5**
Median (IQR)	**5.0 (4.0-8.0)**	**5.0 (4.0-6.0)**	**5.0 (4.0-9.0)**	**6.0 (4.0-8.0)**	**5.0 (4.0-7.0)**
**Initial vancomycin trough level (mg/L) **					
Mean ± SD	**9 ± 5.2**	**9 ± 4.9**	**9 ± 5.5**	**8.7 ± 5.2**	**9.3 ± 5.2**
**Daily vancomycin dose (mg/kg/day) **					
Mean ± SD	**45.8 ± 14.1**	**44.2 ± 14.3**	**49.2 ± 13.7**	**43 ± 13.4**	**48.5 ± 14.7** **∗**
**Total LOS (days) **					
Mean ± SD	**24.7 ± 37.5**	**13.0 ± 13.5**	**41.8 ± 49.2**	**25.6 ± 39.8**	**10.3 ± 9.2** **∗**
Median (IQR)	**11.0 (7.0-21.8)**	**8.5 (6.0-14.0)**	**19.5 (10.0-60.0)**	**12.0 (8.0-21.0)**	**7.0 (6.0-12.0)**

*∗*Difference is highly significant (p<0.001).

**Table 5:** Cases of acute kidney injury.

Case	Treatment Regimen	Age	Gender	Dose of vancomycin (mg/kg/day)	Frequency	Trough (mg/L)	Duration of treatment	Use of concomitant nephrotoxic medication
1	Vancomycin and PTZ	14	M	50	Every 6 hours	8	23 days	Yes (gentamicin)
2	Vancomycin and PTZ	7	F	60	Every 6 hours	No Trough	5 days	Yes (Acyclovir)
3	Vancomycin and PTZ	6	M	60	Every 8 hours	11.6	9 days	No
4	Vancomycin and Ceftriaxone	13	F	75	Every 8 hours	27.5	5 days	No

## Data Availability

The data used to support the findings of this study are restricted by the KAIMRC in order to protect patient privacy. Data are available from KAIMRC for researchers who meet the criteria for access to confidential data.

## Abbreviations

PTZ: Vancomycin and piperacillin-tazobactam
AKI: Acute kidney injury
MRSA: Methicillin-resistant Staphylococcus aureus
KASCH: King Abdullah Children Specialized Hospital
KDIGO: Kidney Disease: Improving Global Outcomes
SCr: Serum creatinine
URTI: Upper respiratory tract infection
G5PD: Glucose-6-Phosphate Dehydrogenase
GERD: Gastroesophageal reflux disease
BUN: Blood urea nitrogen
LOS: Length of stay.

## References

[1] Cataldo M. A., Tacconelli E., Grilli E., Pea F., Petrosillo N. (2012). Continuous versus intermittent infusion of vancomycin for the treatment of gram-positive infections: Systematic review and meta-analysis. *Journal of Antimicrobial Chemotherapy*.

[2] Hussain S., Syed S., Baloch K. (2010). Electrolytes imbalance: A rare side effect of piperacillin/tazobactam therapy. *Journal of the College of Physicians and Surgeons Pakistan*.

[3] Bennett J. E., Dolin R., Blaser M. J. (2014). *Principles and Practice of Infectious Diseases*.

[4] Magill S. S., Edwards J. R., Beldavs Z. G. (2014). Prevalence of antimicrobial use in us acute care hospitals, may-september 2011. *Journal of the American Medical Association*.

[5] Liu T. J., Lam J. P. (2012). Piperacillin-tazobactam-induced acute interstitial nephritis with possible meropenem cross-sensitivity in a patient with osteomyelitis. *American Journal of Health-System Pharmacy*.

[6] Lodise T. P., Patel N., Lomaestro B. M., Rodvold K. A., Drusano G. L. (2009). Relationship between initial vancomycin concentration-time profile and nephrotoxicity among hospitalized patients. *Clinical Infectious Diseases*.

[7] Bosso J. A., Nappi J., Rudisill C. (2011). Relationship between vancomycin trough concentrations and nephrotoxicity: A prospective multicenter trial. *Antimicrobial Agents and Chemotherapy*.

[8] Burgess L. D., Drew R. H. (2014). Comparison of the incidence of vancomycin-induced nephrotoxicity in hospitalized patients with and without concomitant piperacillin-tazobactam. *Pharmacotherapy*.

[9] Gomes D. M., Smotherman C., Birch A. (2014). Comparison of acute kidney injury during treatment with vancomycin in combination with piperacillin-tazobactam or cefepime. *Pharmacotherapy*.

[10] Navalkele B., Pogue J. M., Karino S. (2017). Risk of acute kidney injury in patients on concomitant vancomycin and piperacillin-tazobactam compared to those on vancomycin and cefepime. *Clinical Infectious Diseases*.

[11] Peyko V., Smalley S., Cohen H. (2017). Prospective comparison of acute kidney injury during treatment with the combination of piperacillin-Tazobactam and vancomycin versus the combination of cefepime or meropenem and vancomycin. *Journal of Pharmacy Practice*.

[12] Karino S., Kaye K. S., Navalkele B. (2016). Epidemiology of acute kidney injury among patients receiving concomitant vancomycin and piperacillin-tazobactam: Opportunities for antimicrobial stewardship. *Antimicrobial Agents and Chemotherapy*.

[13] Moenster R. P., Linneman T. W., Finnegan P. M., Hand S., Thomas Z., McDonald J. R. (2014). Acute renal failure associated with vancomycin and *β*-lactams for the treatment of osteomyelitis in diabetics: piperacillin-tazobactam as compared with cefepime. *Clinical Microbiology and Infection*.

[14] Hammond D. A., Smith M. N., Painter J. T., Meena N. K., Lusardi K. (2016). Comparative incidence of acute kidney injury in critically ill patients receiving vancomycin with concomitant piperacillin-tazobactam or cefepime: a retrospective cohort study. *Pharmacotherapy*.

[15] Pratt J. A., Stricherz M. K., Verghese P. S., Burke M. J. (2014). Suspected piperacillin-tazobactam induced nephrotoxicity in the pediatric oncology population. *Pediatric Blood & Cancer*.

[16] Ibach B. W., Henry E. D., Johnson P. N. (2016). Acute kidney injury in a child receiving vancomycin and piperacillin/tazobactam. *The Journal of Pediatric Pharmacology and Therapeutics*.

[17] McKamy S., Hernandez E., Jahng M., Moriwaki T., Deveikis A., Le J. (2011). Incidence and risk factors influencing the development of vancomycin nephrotoxicity in children. *Journal of Pediatrics*.

[18] Le J., Ny P., Capparelli E. (2015). Pharmacodynamic characteristics of nephrotoxicity associated with vancomycin use in children. *Journal of the Pediatric Infectious Diseases Society*.

[19] Knoderer C. A., Nichols K. R., Lyon K. C., Veverka M. M., Wilson A. C. (2014). Are elevated vancomycin serum trough concentrations achievedwithin the first 7 days of therapy associatedwith acute kidney injury in children?. *Journal of the Pediatric Infectious Diseases Society*.

[20] McQueen K. E., Clark D. W. (2016). Does Combination Therapy With Vancomycin and Piperacillin-Tazobactam Increase the Risk of Nephrotoxicity Versus Vancomycin Alone in Pediatric Patients?. *The Journal of Pediatric Pharmacology and Therapeutics*.

[21] Downes K. J., Cowden C., Laskin B. L. (2017). Association of acute kidney injury with concomitant vancomycin and piperacillin/tazobactam treatment among hospitalized children. *JAMA Pediatrics*.

[22] Masoumi H. T., Khataminia M., Hadjibabaie M. PS-035Evaluation of pharmacist intervention on vancomycin dosing and nephrotoxicity prevention.

[23] Marquis K. A., DeGrado J. R., Labonville S., Kubiak D. W., Szumita P. M. (2015). Evaluation of a pharmacist-directed vancomycin dosing and monitoring pilot program at a tertiary academic medical center. *Annals of Pharmacotherapy*.

[24] Smith A. P., Millares-Sipin C. A., James M., Cohen H. (2016). Impact of a pharmacist-initiated vancomycin monitoring program. *Consultant Pharmacist*.

